# The association between flexural eczema, rhinitis, asthma and atopic sensitization in Albanian schoolchildren

**DOI:** 10.1186/2045-7022-5-S1-O13

**Published:** 2015-03-11

**Authors:** Erjola Piluri Ziu, Eris Mesonjesi, Blerina Bregu, Maria Zoto, Alfred Priftanji

**Affiliations:** 1 American Hospital, Department of Allergy and Clinical Immunology, Tirana; Albania; 2 University Hospital Center "Mother Teresa", Department of Allergy and Clinical Immunology, Tirana, Albania

## Background

Atopic dermatitis (AD) is a common, relapsing, inflammatory skin disease that primarily affects young children, in which allergic sensitization is present in only a part of patients. Patients with AD may develop allergic rhinitis and asthma at certain ages. In Albania, prior to the ISAAC Phases survey, very little was known about the prevalence of allergic disorders. To give a recent view of the situation was conducted ALB- ISSAC study during the school year 2011-2012 in Tirana, Albania.

## Methods

ALB- ISAAC study was based on ISAAC protocols. Symptoms core and risk factor questionnaires, allergen skin prick tests, examination for visible flexural dermatitis were completed in a schedule according to the study protocol. In August 2011, a randomized sample of schools was chosen until a predefined sample of 3000 children for each of three age groups; 6-7, 10-11 and 13-14 years old were achieved.

## Results

In 6-7 years old the frequency of eczema ever and visible flexural dermatitis were respectively 1.8% and 1.3%, in 13-14 years old were 2.5% and 0.6%, and in 10-11 years old were 1.7% and 1.3%. The prevalence of sensitization for any allergen was 24.3 % (258/1093). Wheeze ever (OR= 1.50, 95% CI 1.04- 2.17), asthma ever (OR= 2.72, 95% CI 1.36-5.43) and hay fever ever (OR= 2.24, 95% CI 1.24-4.07) were more common among the children with positive skin prick test. While, wheeze past 12 months (OR= 1.71, 95% CI 0.98-2.98) and Nose/eyes past 12 months (OR= 1.74, 95% CI 0.96-3.15) were more frequent among 10-11- years -old with positive SPT, but with no statistical significance. Visible flexural dermatitis was three times more frequent among 10-11-year-olds with positive skin prick tests in 2011-2012 (OR =3.16, 95% CI 1.47-6.82).

## Discussion

The prevalence of eczema symptoms, and visible flexural dermatitis at examination, already below the global average in 1995, has remained stable. Alb-ISAAC study found a significant increase in the prevalence of atopic sensitization. Our study found that, the visible flexural dermatitis, wheeze ever, hay fever ever, asthma ever were more frequent among 10-11 years old with positive skin prick test. Different authors tend to identify different phenotypes of atopic dermatitis and even different phenotype of atopy to evaluate the progression toward rhinitis and asthma. It seems that only the allergic extrinsic form follow the distribution and risk pattern that have been assign to asthma and hay fever.

